# Somatosensory evoked potentials of the tibial nerve during the surgical decompression of thoracolumbar intervertebral disk herniation in dogs

**DOI:** 10.3389/fvets.2022.976972

**Published:** 2022-09-15

**Authors:** Seiichi Okuno, Hirotaka Katahira, Kensuke Orito

**Affiliations:** ^1^Laboratory of Physiology II, School of Veterinary Medicine, Azabu University, Sagamihara, Japan; ^2^Veterinary Clinic of Neurology, Isesaki, Japan; ^3^Laboratory of Environmental Biology, School of Life and Environmental Science, Azabu University, Sagamihara, Japan

**Keywords:** somatosensory evoked potential, IVDH, hemilaminectomy, dog, intraoperative monitoring

## Abstract

This study aimed to identify the impact on spinal cord integrity and determine the electrophysiological safety level during surgery for thoracolumbar intervertebral disk herniation in dogs. A total of 52 dogs diagnosed with thoracolumbar intervertebral disk herniation were enrolled. The tibial nerve somatosensory evoked potential elicited on the scalp by stimulation of the tibial nerve was recorded before and during hemilaminectomy. Both the amplitude and latency of the somatosensory evoked potential were periodically registered, and the percentage changes from the pre-operative control values (amplitude rate and latency rate) were calculated. When the multifidus muscles were retracted after removal from the spinous processes and vertebrae, the somatosensory evoked potential amplitude rate decreased in all dogs, while the latency rate increased in 33 dogs examined. The amplitude rate remained unchanged during the halting procedure, loosening retraction, and hemilaminectomy. After removing the disc material from the spinal canal, the amplitude rate was increased. The somatosensory evoked potential latency increased when the multifidus muscles were retracted and shortened after multifidus muscles closure in four cases. The outcome of all cases showed improvement in clinical signs 7 days after operation. Spinal cord conduction is impaired by retraction of the multifidus muscles and improved by removal of disk materials. Maintaining intraoperative SEP amplitudes above 50% of control may help avoid additional spinal cord injury during surgery. Since we have no case that worsened after the surgery, however, further studies are necessary to confirm this proposal.

## Introduction

Thoracolumbar intervertebral disc herniation (IVDH) is a common disorder in dogs that mainly affects the chondrodystrophic breeds. Backache and neurological disorders in the pelvic limbs are clinical signs of thoracolumbar disk disease, and urinary dysfunction and loss of pain sensation of the pelvic limbs are observed in cases with more severe lesions. Decompression surgery, such as hemilaminectomy, is performed on dogs with neurological deficits and evidence of severe spinal cord compression, with most commonly reported intraoperative complication being insufficient removal of disk materials (1). However, the effects of surgical procedures on spinal cord integrity have not yet been verified and no method has been established to assess spinal cord function during surgery or determine its safety level in veterinary medicine.

Somatosensory evoked potentials (SEPs) are brain and spinal cord responses induced by stimulation of peripheral sensory nerves in the limbs or cranial nerves with sensory function. SEP reflects transmission of the afferent volley from the peripheral sensory nerves to the primary somatosensory cortex, through the myelinated dorsal columns and the medial lemniscal pathways. Cortical recording of SEP is considered to be a field potential arising from the cerebral cortex (2). In humans, SEP has been utilized for evaluation of the spinal cord somatosensory conductive function during spinal surgeries (3–5), and intraoperative SEP monitoring was reported to be useful for reduction of post-operative paraplegia by more than 50–60% (4, 6). In humans, intraoperative SEP amplitude of 40% or less caused post-operative neurological deficit (3), and the absence of SEP during spinal surgery led to new complication or neurological loss of function (4, 6). Therefore, a 50% decrease in SEP amplitude during spinal surgeries is reportedly the alarm threshold for spinal cord damage in humans (4, 7).

In dogs, SEP is recorded when electrical stimulation is applied to the peripheral sensory nerve or cranial nerve with sensory function (8–10). Studies have reported that SEP amplitude in dogs is decreased by spinal cord compression and blocked blood supply to the spinal cord (11–13), suggesting that intraoperative SEP examination is a useful tool for monitoring spinal cord integrity during spinal surgery in veterinary medicine. Notably, we previously reported the usefulness of SEP monitoring during cervical operation in dogs (14). However, to the best of our knowledge, there are no reports of intraoperative SEP monitoring being utilized during a thoracolumbar spinal operation in veterinary medicine.

In this study, we measured the amplitude and latency rates of SEP and evaluated spinal cord integrity during each surgical procedure of hemilaminectomy. Based on the results of this study and previous knowledge of human medicine, we propose that maintaining intraoperative SEP amplitudes >50% of control may help avoid additional spinal cord injury during surgery.

## Materials and methods

### Animals

This study was conducted in a prospective manner. All dogs enrolled in this study were handled in compliance with the Azabu University Animal Experiment Guidelines (April 2000) (17114-2, 191205-5). Dogs were eligible for enrollment after obtaining informed consent from their owners. All surgery was performed under isoflurane anesthesia.

Dogs with thoracolumbar IVDH of clinical grade 2, 3, and 4 that were determined by conventional neurological examination and MRI, and stable SEP was obtained in the pre-operative examination were included in this study. All the cases were diagnosed as Hansen Type 1 intervertebral disk extrusion. Fifty-two dogs (27 males and 25 females) were enrolled between May 2017 and March 2020. The cases showed clinical grade 5 were not enrolled in this study because cortical SEP could not be elicited as reported previously (15). The cases included 41 miniature Dachshunds, 2 Pomeranians, Papillons, Mixes each, and 1 Chihuahua, Beagle, Boston Terrier, Shiba, and Welsh Corgi Pembroke each. Mean ± SD (range) age and body weight were 7.3 ± 2.9 (2–12) years and 6.1 ± 2.0 (3.1–12.6) kg, respectively. The location of disc herniation differed among the dogs as follows: T12–13 (*n* = 10), T13–L1 (*n* = 16), L1–2 (*n* = 19), and L2–3 (*n* = 7). The cases were classified according to the clinical thoracolumbar IVDH grade, which was assigned based on the severity of clinical signs according to a previously reported classification system as follows (16): grade 2 = ataxia, conscious proprioception deficit, and paraparesis (*n* = 9); grade 3 = paraplegia (*n* = 40); and grade 4 = paraplegia with urinary retention and overflow (*n* = 3).

### Experimental procedures

All dogs were premedicated with atropine sulfate [0.04 mg/kg, subcutaneous (SC); Tanabe Mitsubishi Pharma Corp, Osaka, Japan], midazolam [0.1 mg/kg, intravenous (IV); Dormicum, Maruishi Pharmaceutical Co Ltd, Osaka, Japan], and butorphanol (0.2 mg/kg, IV; Vetorphale, Meiji Seika Pharma Co Ltd, Tokyo, Japan). Anesthesia was induced by alfaxalone (2.0–3.0 mg/kg, IV; Alfaxan. Meiji Seika Pharma Co Ltd, Tokyo, Japan) until the desired effect was obtained and maintained throughout the operation with isoflurane (Isoflu, Zoetis Japan Inc, Tokyo, Japan). Rectal temperature was monitored and body temperature was maintained at between 37.0°C and 38.0°C with heating pads.

**Figure 1:**
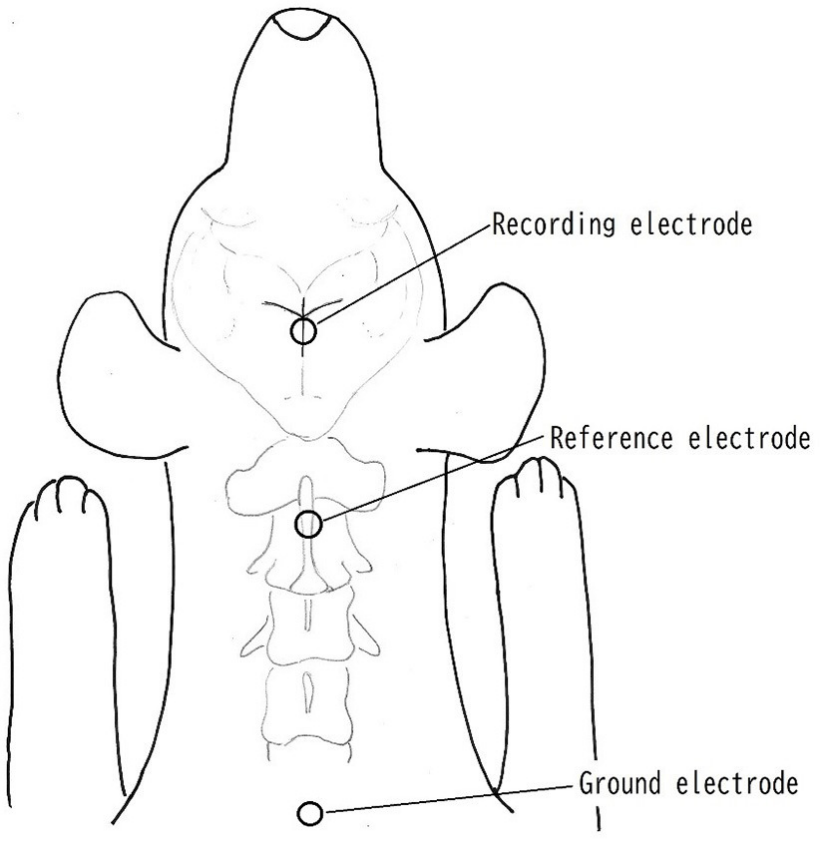
Placement of electrodes. The recording electrode was placed at the juncture of the coronal and sagittal sutures, which were considered to be adjacent to the somatosensory cortex area. The reference electrode was placed on the spinous process of the axis. The ground electrode was placed on the surface of caudal portion of neck.

Surface disk electrodes (Disk Electrode for evoked EEG NE-132B. Nihon Koden Corp, Tokyo, Japan) were used as recording, reference and ground electrodes to check an impedance below 5 K ohms between skin and electrode. The recording electrode was placed at the juncture of the coronal and sagittal sutures, which were considered to be adjacent to the somatosensory cortex area, and the reference and ground electrode was placed on the spinous process of the axis and dorsal surface of caudal portion of neck, respectively, following the method used by Uzuka et al. (9, 14) (Figure 1). The electrical stimuli lasted 0.2 ms, and rectangular waves at a rate of 3 Hz were applied to the tibial nerve on the same side as the operation site. Stainless needles (TERMO needle NN-2525R. TERMO Corp, Tokyo, Japan) were used for the stimulating electrodes, and these were inserted percutaneously and placed immediately proximal to the tarsal joint on the tibial nerve. The cathode was placed proximal ~1 cm from the anode. The stimulating intensity was adjusted with less than 3.6 mA to produce visible small twitch of 5th digit. A total of 200 responses were averaged with high- and low-pass filters at 0.5 and 3,000 Hz, respectively. The recording condition of SEP was 2 μV/division of amplitude and 5 ms/division of sweep speed.

**Figure 2:**
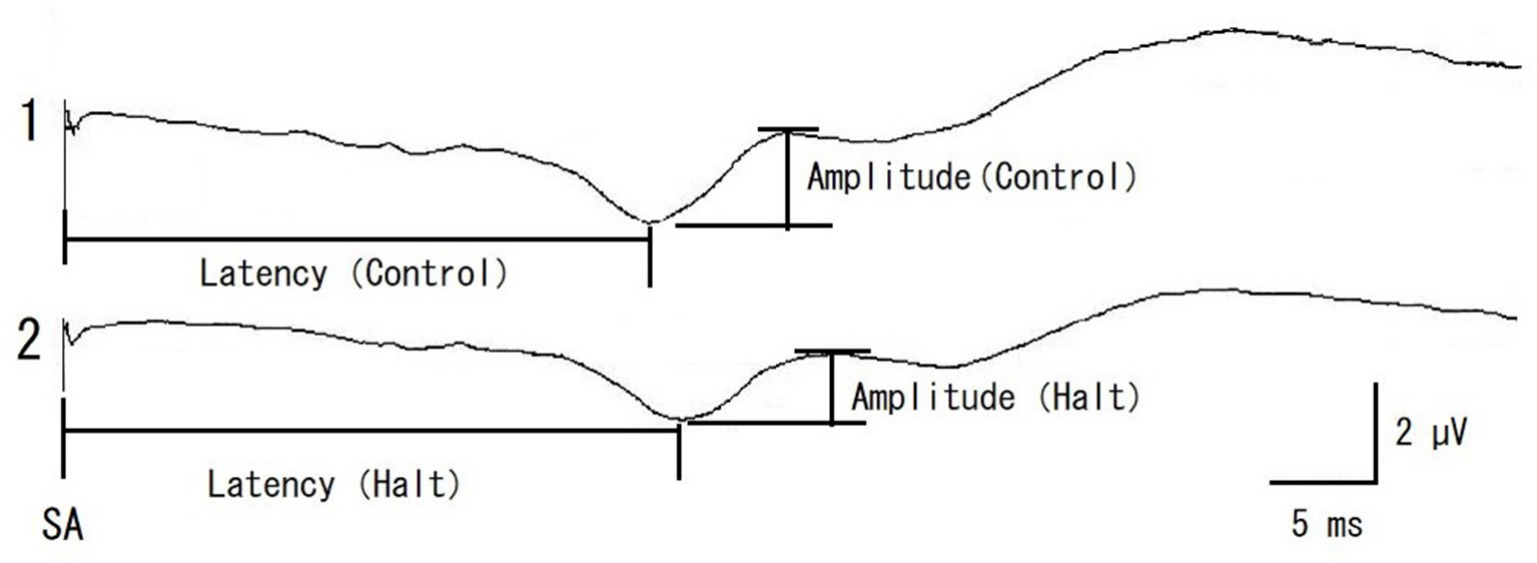
Representative recording of control SEP (pre-operative control, 1) and multifidus muscles retracting (Halt, 2) in a case of 11 years old male miniature dachshund enrolled in this study. The SEP amplitude was determined as the peak-to-peak amplitude of initial positive and next negative waves. The SEP latency was determined from the stimulation artifact (SA) to the peak of initial positive wave. The amplitude rate and latency rate were calculated as follows; The amplitude rate = the amplitude (Halt)/the amplitude (pre-operative control) × 100, The latency rate = the latency (Halt)/the latency (pre-operative control) × 100, respectively.

Somatosensory evoked potential examination was performed as previously described (14). SEP examinations began after induction of anesthesia in the prone position. A stable SEP elicited before the operation served as a control SEP. The SEP amplitude was determined as the peak-to-peak amplitude of the initial positive and subsequent negative waves (Figure 2). The latency from the stimulation artifact (SA) to the peak of the initial positive waves was recorded simultaneously (Figure 2). A system for evoked potentials (Neuropack MEB-9404, Nihon Kohden Corp, Tokyo, Japan) was used to provide electrical stimulations as well as measure the amplitude and latency.

The surgical procedure was performed as follows. The skin and superficial tissues were incised lateral to the midline. The dorsal fascia was incised near each spinous process. The multifidus muscles were peeled off from the spinous and articular processes using an elevator. The multifidus muscles were retracted laterally using Gelpi retractors. The articular processes and vertebral laminae were removed using rongeurs. Disk materials compressing the spinal cord were removed using forceps or curettes.

**Figure 3:**
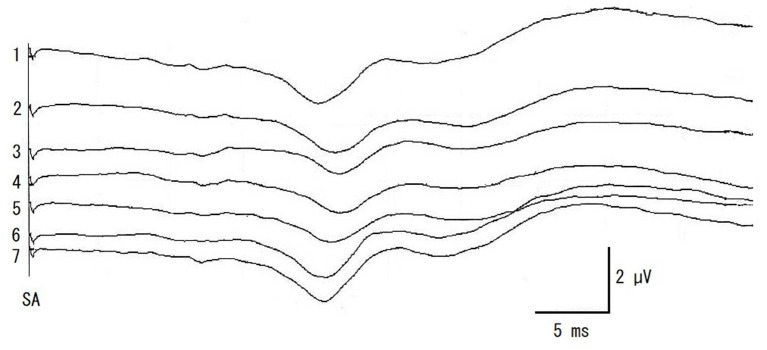
The recordings of intraoperative SEP monitoring in a case of Figure 2. 1: pre-operative control, 2: retracting multifidus muscles, 3: halting surgical procedure and multifidus muscles loosened, 4: hemilaminectomy, 5: removing the disk materials from the spinal canal, 6: closing multifidus muscles, 7: end of the surgery. SA, electrical stimulation artifact.

Somatosensory evoked potential recordings were performed once for each surgical procedure during surgery, starting at multifidus muscles retraction (Retract). When a decrease in amplitude was recognized, surgical manipulations were halted and the retraction of the multifidus muscles was loosened. SEP was then recorded once (Halt). After this recording of SEP (about 2 min), the operation was continued and SEP were recorded at articular processes and laminae resection (Hemilami), intervertebral disk material removal (Remove), and muscle closing (Close). SEP was also recorded at the end of the surgery (End). Representative SEPs and measured amplitude and latency of control and Retract are shown in Figure 2. And the result of SEP recording is shown in Figure 3.

The SEP amplitude is negatively correlated with body size in dogs (10). Low body temperature and low blood pressure cause slowing of conduction velocity and reduced amplitude of SEP, respectively (4, 6). Therefore, the amplitude rate and latency rate were used in this study to evaluate the changes in SEP during surgical procedures of various bleeds.

Amplitude and latency rate were calculated as follows:


$$\begin{array}{l} \text{Amplitude rate }\left(\%\right)=\;\frac{\text{Recorded SEP amplitude}}{\text{Control SEP amplitude}}\times 100 \end{array}$$

$$\begin{array}{l} \text{Latency rate }\left(\%\right)=\;\frac{\text{Recorded SEP latency}}{\text{Control SEP latency}}\times 100 \end{array}$$

### Statistical analyses

Data are expressed as mean ± SD (range). Amplitude and latency rates were compared between surgical procedures as a fixed effect by fitting a linear mixed model (LMM), considering individual differences as a random effect (17). The significance of each constructed model was evaluated by a likelihood ratio test based on an approximate chi-square distribution against the null model deviance. When the model significance was confirmed, the estimated coefficients for the mean rate at each surgical procedure were compared using Tukey's *post-hoc* honestly significant difference tests. These analyses were performed using the *lme4* and *multcomp* packages in the R 4.0.3 (18). Statistical significance was set at *p* < 0.05.

### Clinical outcome evaluation

Clinical outcome evaluation of all cases was performed 7 days after operation using the conventional neurological examinations same as pre-operative examination. An improvement was determined by the following changes in clinical signs: disappearance of neurological deficit in cases of grade 2, and walking without any assistance in cases of grade 3 and 4.

## Results

Stable SEPs were recorded throughout the surgery and examined for all cases in this study. Isoflurane concentration was stable and any additional agents was not used throughout operation in all cases. There were no cases in which clinical signs deteriorated after surgery. All of the cases of grade 2 showed no neurological deficit and all patients of grade 3 and 4 were able to walk without any assistance within 7 days after surgery.

When the multifidus muscles were removed from the vertebrae and retracted, the SEP amplitude decreased in all cases, and its latency increased in 33 dogs examined (Figure 2). The deviances in both constructed models for the amplitude and latency rates differed significantly from those in the null models (χ^2^ = 191.45, df = 5, *p* < 0.001 in amplitude; χ^2^ = 30.01, df = 5, *p* < 0.001 in latency). The estimated intercept (i.e., the relative difference in the process of the retracting multifidus muscles) in the amplitude rate was significantly lower (69% with the mean value, *t* = 36.99, *p* < 0.001) than in the pre-operative control (Table 1), while in the latency rate was significantly higher (104% with the mean value, *t* = 186.91, *p* < 0.001) than in the control (Table 2). The standard deviations of the intercept variability caused by individual differences were 10.05 in the amplitude rate and 3.62 in the latency rate.

**Table 1:** Number of dogs in each range of amplitude rate of six surgical procedures.

**Amplitude rate (% of pre-operative control values)**	**Retract^a^**	**Halt^a, b^**	**Hemilami^a, b^**	**Remove^b^**	**Close^c^**	**End^c^**
≥140	0	0	0	0	0	0
130–140	0	0	0	1	1	1
120–130	0	0	0	0	0	2
110–120	0	0	0	1	3	4
100–110	0	1	1	1	8	11
90–100	1	2	1	2	8	8
80–90	6	7	8	12	14	10
70–80	16	20	21	18	13	12
60–70	17	17	17	16	4	3
50–60	12	5	4	1	1	1
<50	0	0	0	0	0	0
Mean ± SD (% of pre-operative control values)	69 ± 11	73 ± 11	73 ± 10	77 ± 14	88 ± 16	92 ± 17
Range (% of pre-operative control values)	52–93	54–100	54–100	58–132	58–132	58–132

Retract, retracting multifidus muscles; Halt, halting surgical procedure and multifidus muscles loosened; Hemilami, hemilaminectomy; Remove, removing the disk materials from the spinal canal; Close, closing multifidus muscles; End, end of the surgery. Procedures with different letters indicate significant difference (p < 0.05).

**Table 2:** Number of dogs in each range of latency rate of six surgical procedures.

**Latency rate (% of pre-operative control values)**	**Retract^a, b, c^**	**Halt^a^**	**Hemilami^a^**	**Remove^a, b^**	**Close^b, c^**	**End^c^**
≥120	0	0	0	0	0	0
110–120	2	3	3	2	1	1
100–110	50	49	49	49	47	45
90–100	0	0	0	1	4	6
<90	0	0	0	0	0	0
Mean ± SD (% of pre-operative control values)	104 ± 4	104 ± 4	104 ± 4	104 ± 4	103 ± 4	103 ± 4
Range (% of pre-operative control values)	100–119	100–119	100–119	98–117	93–114	93–114

Retract, retracting multifidus muscles; Halt, halting surgical procedure and multifidus muscles loosened; Hemilami, hemilaminectomy; Remove, removing the disk materials from the spinal canal; Close, closing multifidus muscles; End, end of the surgery. Procedures with different letters indicate significant difference (p <0.05).

Although amplitude rate in End was still lower than pre-operative control in 34 cases, the *post-hoc* test comparing the pairs of the estimated means, supported the tendency of increasing amplitude rates during the surgical procedure (Table 1). In one case the amplitude rate decreased in Retract, increased in Halt, and then decreased again in Hemilami. It was, however, still higher than that in Retract. Therefore, surgical manipulations were not halted again. Although the mean amplitude rates in Halt and Hemilami tended to be higher than those in Retract, significant differences were not observed (4.49%, *z* = 2.60, *p* = 0.097 and 4.67%, *z* = 2.70, *p* = 0.075, respectively). The mean amplitude rates in Remove, Close, and End were higher (8.63%, 19.21%, and 22.98%, respectively; *p* < 0.001) than those in Retract. The mean amplitude rates in Halt, Hemilami, and Remove also differed from those of Close and End (10.58–22.98% lower, *p* < 0.001 in each comparison). For latency rates, the estimated mean values increased in Retraction, Halt, Hemilami, and Remove. Latency rates decreased from these Halt and Hemilami steps to Close and End (1.03–1.47%, *p* < 0.022 in each comparison) and from Remove to End (1.12%, *p* = 0.01) (Table 2).

## Discussion

The effects of surgical procedures of hemilaminectomy on spinal cord integrity were verified using intraoperative SEP monitoring. The multifidus muscles retraction affects spinal cord conductivity. It is suggested that SEP could be the method to assess spinal cord function during surgery in veterinary medicine.

Somatosensory evoked potentials correspond to spinal cord and cortical sensory responses evoked following electrical stimulation of a peripheral sensory nerve or cranial nerves with sensory function; therefore, SEP reflects the function of sensory pathways in the spinal cord and brainstem for cranial nerve studies. Cortical recording of SEP are affected by various factors such as anesthetic agents, nerve stimulate rate, body size (length of pathway), physiological condition and spinal cord integrity of patient (2, 4, 10). Although cortical evoked potentials are attenuated by inhalation anesthetics, isoflurane has little effect on SEP even when bolus doses are administered to obtain a high anesthetic effect during surgery (4, 6). The effect of premedication and induction agent of anesthesia on SEP parameters was unclear. Stimulation frequency affects SEP amplitude. For example, higher amplitude SEP could be obtained by lower frequency stimulation. Because it takes longer time to record, the lower frequency is not feasible for recording of SEP during surgery. Stimulus intensity was determined by small twitch of 5th digit that was induced by slight activation of motor nerves. Activation threshold of the sensory nerve is much lower than that of the motor nerve. Thus, almost all sensory nerve could be activated when the motor nerve was activated slightly (19).

The SEP amplitude evoked by stimulation of the sciatic nerve decreased when compression was applied to the spinal cord, and recovered after release of the applied compression (11). In an experimental study of canine spinal cord ischemia, SEP elicited by stimulation of the sciatic nerve disappeared within a mean ischemic time of 12.4 min. When reperfusion was instituted immediately following the abolition of SEP, SEP recovered and there were no neurologic sequelae. However, when occlusion was maintained for an additional 15 min following abolition of SEP, spastic paraplegia with corresponding histological changes indicative of spinal cord infarction developed (11). Thus, SEP amplitude may be decreased by compression or ischemia of the spinal cord. The release of the compression and the ischemia are important to restore SEP amplitude. In more than 50% of patients, reduction of SEP amplitude and prolongation of its latency after retracting multifidus muscles were not recovered to the pre-operative control values even at the end of surgical procedure. However, all of the patients could walk without any assistance within 7 days after surgery, suggesting that neurological function has been recovered day by day.

In this study, the SEP amplitude rate decreased and the SEP latency rate increased both when the multifidus muscles were removed from the vertebrae and retracted. Therefore, this may indicate that these procedures induce compression of the spinal cord and/or a decrease in blood supply to the spinal cord under IVDH. The SEP amplitude rate continued to decrease and its latency rate continued to increase even after halting surgical manipulation and loosening the muscle retraction and hemilaminectomy, suggesting that perfusion of the spinal cord may not recover sufficiently during these procedures. Removal of the disk material from the spinal canal may release compression from the spinal cord and blood flow recovery, as the SEP amplitude recovers after this procedure. Further studies, however, are necessary to prove this hypothesis The results of this study indicate that to remove the disk materials that compress the spinal cord may be required to improve spinal cord function. However, surgical manipulation to remove disk materials from the spinal canal poses the risk of compression of the spinal cord. Therefore, it is important to monitor spinal cord function to avoid additional spinal cord compression during surgery.

In this study, the minimal SEP amplitude rate during the surgery was 52% of the control, and the clinical signs of all dogs were improved within 7 days after the surgery. A previous study reported that the safety limit of SEP amplitude was 50% of the control (13). A 50% of the control in SEP amplitude is generally considered alarming in humans (4). In this study, there were no cases SEP amplitude being less than 50% of control. And no cases worsened clinical signs. Therefore, alarm level of neurological deficit of spinal cord cannot be determined. We propose here that maintaining intraoperative SEP amplitudes above 50% of control may help avoid additional spinal cord injury during surgery. Further studies are necessary to confirm this proposal.

There are some limitations in this study. Although intraoperative monitoring of SEP has the potential to increase the safety of spine and spinal cord surgery, SEP can assess only sensory pathway from peripheral sensory nerve to the sensory cortex. Dysfunction of the motor pathway from motor cortex to the muscles cannot be evaluated by SEP. Dysfunction of the motor pathway can be evaluated by motor evoked potential method, utilizing a transcranial magnetic stimulation (20, 21). Because SEP could not be obtained in dogs with grade 5, neurological function cannot be evaluated in such patients.

## Conclusions

Intraoperative SEP is useful for monitoring spinal cord integrity during surgical procedure of spine or spinal cord. The retraction of the multifidus muscles under the condition of compressive disk materials would especially cause a decrease in blood flow and/or compression of the spinal cord. Therefore, additional compression of the spinal cord should be avoided during surgery. Maintaining an SEP amplitude rate above 50% of control is important to avoid excessive spinal cord injury during surgery.

## Data availability statement

The raw data supporting the conclusions of this article will be made available by the authors, without undue reservation.

## Ethics statement

The animal study was reviewed and approved by Azabu University Animal Experimentation Committee. Written informed consent was obtained from the owners for the participation of their animals in this study.

## Author contributions

SO wrote the majority of the manuscript, performed the surgeries, and SEP examinations. HK wrote statistical analysis parts and performed the statistical analysis. SO and KO designed the study. All authors took part in editing the manuscript. All authors contributed to the article and approved the submitted version.

## Conflict of interest

The authors declare that the research was conducted in the absence of any commercial or financial relationships that could be construed as a potential conflict of interest.

## Publisher's note

All claims expressed in this article are solely those of the authors and do not necessarily represent those of their affiliated organizations, or those of the publisher, the editors and the reviewers. Any product that may be evaluated in this article, or claim that may be made by its manufacturer, is not guaranteed or endorsed by the publisher.

## References

[1] Sharp N Wheeler S. Thoracolumbar disk disease. In: Sharp N, Wheeler S, editors. Small Animal Spinal Disorders: Diagnosis and Surgery. 2nd ed. St Louis, MO: Mosby Elsevier (2005). p. 121–35. 10.1016/B978-0-7234-3209-8.50012-1

[2] Holliday TA. Electrodiagnostic examination. Somatosensory evoked potentials and electromyography. Vet Clin North Am Small Anim Pract. (1992) 22:833–57. 10.1016/S0195-5616(92)50079-1 1641920

[3] Fisher RS Raudzens P Nunemacher M. Efficacy of intraoperative neurophysiological monitoring. J Clin Neurophysiol. (1995) 12:97–109. 7896914

[4] Nuwer MR. Spinal cord monitoring. Muscle Nerve. (1999) 22:1620–30. 10567073 10.1002/(sici)1097-4598(199912)22:12<1620::aid-mus2>3.0.co;2-1

[5] Pelosi L Lamb J Grevitt M Mehdian SMH Webb JK Blumhardt LD. Combined monitoring of motor and somatosensory evoked potentials in orthopedic spinal surgery. Clin Neurophysiol. (2002) 113:1082–91. 10.1016/S1388-2457(02)00027-5 12088704

[6] Kimura J. Intraoperative monitoring. In: Kimura J, editor. Electrodiagnosis in Diseases of Nerve and Muscle. 4th ed. New York, NY: Oxford (2013). p. 573–95. 10.1093/med/9780199738687.003.0021

[7] Zentner J May J. Intraoperative monitoring with somatosensory evoked potentials in neurosurgical operations on the spinal cord. Cent Nerv Syst Trauma. (1987) 4:197–207. 10.1089/cns.1987.4.197 3442816

[8] Vanderzant CW Schott RJ Natale JE Pondo CA D'Alecy LG. Somatosensory evoked potentials of the dog: recording techniques and normal values. J Neurosci Methods. (1989) 27:253–63. 10.1016/0165-0270(89)90087-3 2725007

[9] Uzuka Y Saitoh M Hiramatsu I Nagata T. Studies of the factors affecting the recording of somatosensory evoked potentials induced by tibial nerve stimulation in dogs. J Vet Med Sci. (1995) 57:871–6. 10.1292/jvms.57.871 8593295

[10] Poncelet L Michaux C Balligand M. Influence of body size on tibial nerve somatosensory evoked potentials in dogs. Am J Vet Res. (1993) 54:178–82. 8427464

[11] Dinner DS Lüders H Lesser P Morris HH. Invasive methods of somatosensory evoked potential monitoring. J Clin Neurophysiol. (1986) 3:113–30. 10.1097/00004691-198604000-00002 3084558

[12] Janusz MT Qayumi AK Jamieson WRE Fairholm DJ. Experimental use of somatosensory evoked potential for intraoperative identification of spinal cord blood supply. J Invest Surg. (1997) 10:195–203. 10.3109/08941939709032158 9284004

[13] Shen N Wang S. Monitoring spinal-cord injury intraoperatively and attempting prognosis by cortical somatosensory evoked potentials: experimental study. J Reconst Microsurg. (1998) 14:61–6. 10.1055/s-2007-1006903 9524005

[14] Okuno S Nakamura A Kobayashi T Orito K. Effectiveness of intraoperative somatosensory evoked potential monitoring during cervical spinal operations on animals with spinal cord dysfunction. J Vet Med Sci. (2005) 67:719–22. 10.1292/jvms.67.719 16082122

[15] Poncelet L Michaux C Balligand M. Somatosensory potentials in dogs with naturally acquired thoracolumbar spinal cord disease. Am J Vet Res. (1993) 54:1935–41. 8291776

[16] Sharp N Wheeler S. Patient examination. In: Sharp N, Wheeler S, editors. Small Animal Spinal Disorders: Diagnosis and Surgery. 2nd ed. St Louis, MO: Mosby Elsevier (2005). p. 19–33. 10.1016/B978-0-7234-3209-8.50006-6

[17] Zuur AF Ieno EN Walker N Saveliev AA Smith GM. Mixed effects modeling for nested data. In: Zuur AF, Ieno EN, Walker N, et al., editors. Mixed Effects Models and Extensions in Ecology With R. New York, NY: Springer (2009). p. 101–42. 10.1007/978-0-387-87458-6_5

[18] R Core Team. A Language and Environment for Statistical Computing. Vienna: R Foundation for Statistical Computing (2020).

[19] Kimura J. Somatosensory evoked potential. In: Kimura J, editor. Electrodiagnosis in Diseases of Nerve and Muscle. 4th ed. New York, NY: Oxford (2013). p. 477–524. 10.1093/med/9780199738687.003.0019

[20] Poma R Parent JM Holmberg DL Partlow GD Monteith G Sylvestre AM. Correlation between severity of clinical signs and motor evoked potentials after transcranial magnetic stimulation in large-breed dogs with cervical spinal cord disease. J Am Vet Med Assoc. (2002) 221:60–4. 10.2460/javma.2002.221.60 12420825

[21] Martin-Vaquero P de Costa RC. Transcranial magnetic motor evoked potentials in Great Danes with and without clinical signs of cervical spondylomyelopathy: association with neurological findings and magnetic resonance imaging. Vet J. (2014) 201:327–32. 10.1016/j.tvjl.2014.05.035 24929532 PMC4160397

